# Pre‐culture with transferrin‐Fe^3+^ before in vitro maturation improves the developmental competence of porcine oocytes matured in vitro

**DOI:** 10.1002/rmb2.12529

**Published:** 2023-08-04

**Authors:** Shingo Tonai, Tomoya Nakanishi, Manami Yamaoka, Asako Okamoto, Masayuki Shimada, Yasuhisa Yamashita

**Affiliations:** ^1^ Graduate School of Comprehensive Scientific Research Prefectural University of Hiroshima Shobara Japan; ^2^ Graduate School of Integrated Sciences for Life Hiroshima University Higashi‐Hiroshima Japan

**Keywords:** in vitro maturation, iron, oocyte, pre‐IVM, transferrin

## Abstract

**Purpose:**

Since the developmental competence of oocytes cultured after in vitro maturation (IVM) is low, it is necessary to improve the IVM method for efficient offspring production. In this study, we revealed that transferrin (TF)‐Fe^3+^ was accumulated in follicular fluid with increasing the follicular diameter, and that TF receptor (TFR1) was localized in granulosa cells of pig. Thus, we hypothesized that TF‐Fe^3+^ would be a factor in the induction of developmental competence of porcine oocytes.

**Methods:**

To mimic the follicular development environment, cumulus–oocyte complexes (COCs) were cultured in pre‐IVM medium (low dose of FSH) without or with Holo‐TF (monoferric or diferric TF) or Apo‐TF (non‐iron bond TF). After pre‐IVM without or with Holo‐TF, COCs were cultured in IVM medium (high dose of FSH and EGF) without or with Holo‐TF.

**Results:**

Cultivation with Holo‐TF increased the expression of follicular development maker (*Cyp19a1* and *Ccnd2*), E2 production, and proliferative activity of cumulus cells, whereas cultivation with Apo‐TF did not show these positive effects. The treatment with Holo‐TF during pre‐IVM, but not during IVM, dramatically induced oocyte maturation with increasing the blastocyst rate.

**Conclusion:**

We succeeded in showing for the first time that the cultivation with Holo‐TF in pre‐IVM can produce embryos in pig with high efficiency.

## Graphical abstract

Since transferrin and transferrin receptor is contained in follicular fluid and granulosa/cumulus cells of middle‐sized antral follicle, we investigated the effect of TF‐Fe3+ on pre‐IVM during the cultivation of porcine COCs. As the results, the supplementation of TF‐Fe3+ during pre‐IVM before IVM dramatically improved developmental competence of oocytes.

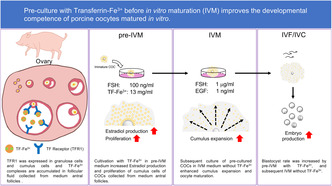

## 1 INTRODUCTION

In vitro maturation (IVM) of cumulus–oocyte complexes (COCs) is an important technique to produce mammalian embryos, including human, mice, bovine, and pigs. In pig, the IVM success rate, measured by the proportion of metaphase II (MII) oocytes is relatively high, but the blastocyst production rate following in vitro fertilization (IVF) is still low compared with that of in vivo‐matured oocytes.^1^, ^2^, ^3^ The developmental competence of in vitro‐matured oocytes is dependent on the IVM culture conditions, suggesting that the current IVM methods is considered technically inadequate. The low blastocyst production rate may be caused by the sudden cultivation with ovulatory environment. Our previous reports showed that developmental competence of oocytes is improved by the cultivation in follicular development environment (pre‐IVM), followed by the cultivation in ovulatory environment (IVM) with transition of hormone environment, including FSH, LH, E2, and P4.^4^, ^5^ Furthermore, other reports also showed that the preincubation before IVM of porcine COCs enhanced developmental competence of oocytes.^6^, ^7^, ^8^ Therefore, it is effective that the cultivation of COCs with pre‐IVM before IVM enhances developmental competence of oocyte.

The mammalian ovary contains follicles at various developmental stages. Follicles are classified as either preantral or antral according to the absence or presence of a cavity. At the antral stage, a few numbers of the small antral follicles selectively develop into full‐grown large antral follicles, but many follicles are closed to atretic follicles. FSH regulates the development of small antral follicle into large ones; it is secreted from the pituitary gland and binds to FSH receptor (FSHR) located in granulosa cells and cumulus cells of small antral follicles. FSH stimulus induces E2 production concomitantly with *Cyp19a1* mRNA expression in granulosa cells. E2 induces *Ccnd2* mRNA expression in granulosa cells of small antral follicles, which induces terminal development to the large antral stage.^9^, ^10^ After follicular development, a transient surge of pituitary gland‐derived LH binds to its receptor (LHCGR) located in granulosa cells, which induce expression of EGF‐like factors and COC expansion‐related factors (Hyaluronan synthase 2; HAS2, Pentraxin3; PTX3, TNF alpha‐induced protein 6; TNFAIP6) in granulosa cells, and these factors then induce COC expansion, oocyte meiotic maturation, and ovulation.^11^, ^12^, ^13^, ^14^, ^15^ In pig, previous reports showed the effectiveness of FSH and EGF supplementation during IVM to induce nuclear maturation and cytoplasmic maturation of oocytes, although even today blastocyst production efficiency is below 20%. Since the dominant follicles are vascularized on the follicle surface,^5^, ^16^ we assumed that the blood‐derived factor(s) enter follicle and induce full maturation in oocyte.

Transferrin (TF), a 78‐kD glycoprotein, is secreted mainly from liver.^17^, ^18^ TF consists of two lobes: one at the N‐terminal region and the other at the C‐terminal region.^19^ Since a lobe can bind to one iron, a TF molecule bind two ferric irons. TF‐bound iron (TF‐Fe^3+^) circulates freely in the serum and in extravascular spaces, and it serves as a source of iron for cells and tissues that are perfused by the systemic circulation. TF receptor 1 (TFR1) functions as a dimer, and each 90‐kD monomer has a single transmembrane‐spanning domain. Most cells modulate iron uptake by regulating the amount of TFR1.^20^ When TFR1 binds to TF‐Fe^3+^, the TF‐Fe^3+^–TFR1 complex is internalized into an endosome, where acidification facilitates the release of Fe^3+^ from TF; the released Fe^3+^ is used in various peripheral tissues, including muscle, heart, kidney, and bone marrow.^21^ During follicular development, although the existence of TF protein was previously shown in granulosa cells,^22^ the physiological effectiveness of TF on COC function has not been reported.

The aim of this study was to characterize the roles of TF‐Fe^3+^ in cumulus cells function and in the developmental competence of oocytes cultured prior to IVM (pre‐IVM) followed by IVM with a physiological concentration of TF‐Fe^3+^ in pig. We firstly investigated the expression of *Tf* and *Tfr1* mRNA in cumulus cells of COCs and the TF concentration in follicular fluid collected from follicles of various sizes. We then examined the effect of supplementation with TF–Fe^3+^ complex to pre‐IVM medium and/or IVM medium in which COCs were cultured on the function of cumulus cells and on developmental competence in pigs.

## 2 MATERIALS AND METHODS

### 2.1 Materials

PBS (−) was dissolved in 0.8% (w/v) NaCl (Fujifilm Wako Chemicals), 0.02% (w/v) KCl (Nacalai Tesque), 0.28% (w/v) NaHPO_4_·12H_2_O (Nacalai Tesque), 0.02% (w/v) KH_2_PO_4_ (Nacalai Tesque), and ultrapure water. PBS (+) was dissolved in 0.1% (w/v) CaCl_2_ (Hayashi Pure Chemical Ind., Ltd), 0.1% (w/v) MgCl_2_·6H_2_O (Katayama Chemical Industries Co, Ltd), 1% (w/v) glucose (Nacalai Tesque), and ultrapure water. PBS was dissolved in 9% (v/v) PBS (−), 10% (v/v) PBS (+), 1% (v/v) PVP (Sigma‐Aldrich), 0.2% (v/v) penicillin streptomycin (Nacalai Tesque), and ultrapure water. Modified NCSU37 (mNCSU37) medium was used as basic medium for pre‐IVM and IVM of porcine COCs.^5^, ^23^, ^24^ Highly purified FSH was a kind gift from the National Hormone and Pituitary Program (National Institute of Diabetes, Digestive and Kidney Diseases; NIDDK). Hypoxanthine (Sigma‐Aldrich) was dissolved in the mNCSU37. Holo‐TF (monoferric or diferric TF; T8158) and Apo‐TF (non‐iron bond TF; T2252) were purchased for Sigma‐Aldrich and were dissolved in the mNCSU37. Fetal calf serum (FCS) was purchased from Invitrogen.

#### 2.1.1 Isolation of porcine COCs and follicle fluid

Porcine ovaries and liver were collected from 5–7‐month‐old prepubertal gilts at a local slaughterhouse and transported within 1 h to the laboratory in 0.85% NaCl containing 1% penicillin–streptomycin mixed solution (Nacalai tesque) at about 30°C. Using a previously described methods,^5^ follicles with diameters of small (1–3 mm), medium (4–7 mm), and large (bigger than 8 mm) antral follicles in the diameter were cut with scissors and were used for immunofluorescence staining. COCs were aspirated from each size follicles by an 18G needle and placed in a 1.5 mL tube. The tube was centrifuged at 100 *g* for 5 min, and the supernatant was stored at −80°C until used for the analysis of TF and iron concentration. Precipitates containing granulosa cells and COCs were placed in a petri dish, and COCs surrounded by more than four layer of unexpanded cumulus cells were collected with a Pasteur pipette under a stereomicroscope. Collected COCs were used for total RNA isolation, pre‐IVM culture, and IVM culture.

### 2.2 Real time RT‐PCR analysis

Total RNA was isolated according to our previous report.^5^ Briefly, total RNA was extracted from COCs and liver using the RNeasy Mini Kit (QIAGEN Sciences) according to the instruction manual and dissolved in nuclease‐free water. The final RNA concentration (5 ng/μL) was determined by absorbance using a microspectrophotometer (Thermo Fisher Scientific).

Reverse transcription was performed as recently described.^16^ Briefly, isolated total RNA (5 ng/μL) was reverse‐transcribed using 500 ng of poly‐dT (Promega) and 0.25 U of avian myeloblastosis virus reverse transcriptase (Promega) for 75 min at 42°C and for 5 min at 95°C.

Real‐time PCR analysis was performed as we described recently.^16^ Briefly, cDNA and primers were added to the KAPA SYBER FAST Universal qPCR kit (Kapa Biosystems) to give a total reaction volume of 15 μL. PCR was then performed using the MiniOpticon Real‐Time PCR Detection Systems (Bio‐Rad). The conditions were as follows: 30 s at 95°C, followed by 40 cycles each of 5 s at 95°C and 45 s at 60°C or 64°C. Specific primers were selected and analyzed as indicated in Table 1. *18s* was used as a control for reaction efficiency and variations in concentrations of mRNA in the original RT reaction. In this study, several housekeeping genes such as *18s* and *Gapdh* were tested. *18s* showed the least variation among treatment groups and was closed to target gene; thus, *18s* was used as the housekeeping gene in this study (data not shown). The results were first normalized to the expression levels of *18s*. To avoid false‐positive signals, dissociation curve analysis was performed at the end of amplification and the PCR products were applied to agarose gel electrophoresis to confirm the size.

**TABLE 1:** Primer sequence and annealing temperatures using quantitative RT‐PCR.

mRNA	Primer sequence	Tm (°C)	Product size (bp)	Accession No.
*18s*	F: 5′‐gag cga aag cat ttg cca ag‐3′	60	107	AF102857
	R: 5′‐ggc atg gtt tat ggt ggg aa‐3′			
*Tf*	F: 5′‐ggg caa aag atc tga agc ag‐3′	64	185	NM_001244653
	R: 5′‐ctc ctc agc aac aca ggt ca‐3′			
*Cyp19a1*	F: 5′‐gct aat tgc agc acc aga ca‐3′	60	195	NM_214429.1
	R: 5′‐ggc tgg tac ctc atg ctc tc‐3′			
*Ccnd2*	F: 5′‐gtt gca gaa ctt gct gac ca‐3′	64	182	NM_214088.1
	R: 5′‐agc ggt cca ggt aat tga tg‐3′			
*Has2*	F: 5′‐ttt gga tgt gtc cag tgc at‐3′	60	152	NM_214053
	R: 5′‐aga ctc agc act cgg ttc gt‐3’			
*Ptx3*	F: 5′‐cag ctc ctg cct ctc act ct‐3′	60	160	NM_001244783.2
	R: 5′‐cca aca ctg cag acc aga ga‐3′			
*Tnfaip6*	F: 5′‐ggc gtg tac ctc aga gaa gc‐3′	60	192	NM_001159607.2
	R: 5′‐tgg ctt tac aat ggg gta gc‐3′			

### 2.3 Quantification of TF in follicular fluid

Follicular fluids were collected into plastic tubes and centrifuged at 2000 *g* for 10 min. After centrifugation, TF concentration of samples was measured by porcine TF EIA Kit (Life Laboratory Company) according to manufacturer instructions.

### 2.4 Measurement of iron concentration in follicular fluid

The fluids collected from small, medium, and large antral follicles were each treated with 6 M HCl to adjust the pH (2, 3), and were put under room temperature for 30 min. Each solution was then centrifuged for 10 min at 1000 *g* and the iron concentration of each supernatant was measured using an iron assay kit (Metallogenics, Co., Ltd.) according to the manufacturer's instructions.

### 2.5 Immunofluorescence staining

Immunofluorescence staining of TFR1 was performed as recently described.^16^ Cut out small, medium, and large antral follicles were fixed by 4% (w/v) paraformaldehyde (PFA; Nacalai tesque)/PBS, stepwise dehydrated by ethanol (Nacalai tesque), clarified in xylene (Fujifilm Wako Chemicals), and embedded in paraffin (Sakura). Embedded follicles were cut at 5 μm using a microtome (Leica RM2125RTS) and placed on the slide. The section was deparaffinized by xylene, stepwise hydrated by ethanol, and washed with PBS (−). The washed section was incubated in 10 mM sodium citrate buffer for 20 min for antigen retrieval and washed with PBS (−). The washed section was then blocked with 5% (w/v) BSA/PBS (−) for 2 h at room temperature, and incubated in diluted TFR1 primary antibody (Abbiotec, Cat. No. 251844, dilution; 1:100, RRID; AB_10643666) by 5% (w/v) BSA/PBS (−) at 4°C overnight. The section was then washed with PBS (−), and reacted with the secondary antibody, an anti‐rabbit IgG F (ab0) fragment Cy3 antibody (Sigma‐Aldrich, Cat. No. C2306, dilution: 1:100, RRID; AB_258792), for 2 h at room temperature in the dark. The reacted section was washed with PBS (−) and stained with DAPI (VECTASHIELD Mounting Medium with DAPI, Vector Laboratories, Inc.). The fluorescence of Cy3 and that of DAPI were detected by a BZ‐X700 microscope (Keyence) using 560 and 360 nm excitation filters, respectively.

Detection of proliferating cell nuclear antigen (PCNA)‐positive cells of cumulus cells in COCs were detected as previously described.^5^ Briefly, cultured COCs were fixed with 4% (w/v) paraformaldehyde (Nacalai tesque) and COCs were permeabilized by 0.5% (v/v) Triton X‐100 in PBS (−) for 30 min at room temperature. COCs were washed with PBS and then blocked with 5% (v/v) BSA (Sigma‐Aldrich) in PBS (−) for 90 min at room temperature. Reaction with the primary antibody applied to the section was then performed with an anti‐PCNA (PC10) mouse monoclonal antibody (Cell Signaling Technology, Inc., Cat. No. #2586, dilution: 1:100, RRID; AB_2160343) for 4 h at room temperature. COCs were washed three times with PBS, after which the secondary antibody reaction was performed with an anti‐mouse IgG F (ab0) fragment Cy3 antibody (Sigma‐Aldrich, Cat. No. C2181, dilution: 1:100, RRID; AB_258785) for 2 h at room temperature in the dark. After washing three times with PBS, the nucleus of COCs in the section were stained with DAPI. The fluorescence signal of PCNA‐positive cells was detected by a BZX‐700 microscopy using a 550 nm excitation filter. In the cumulus cells, the PCNA and DAPI double‐stained cells were counted and then the rate of PCNA‐positive cells was calculated by internal standardization using the number of cells stained with DAPI only. To avoid false‐positive signal, negative control was used oocyte or ovary section incubated vehicle only instead of vehicle including the primary antibody.

### 2.6 Measurement of E2 concentration

Extraction and E2 measurement were performed according to the manufacturer's instruction for the Estrogen ELISA kit (Cayman Chemical). In detail, pre‐IVM culture medium and methanol were mixed at 1:4 and incubated for 10 min at room temperature. After incubation, the solution was centrifuged for 10 min at 2000 *g* and the supernatant was evaporated. The residue was reconstituted in ELISA buffer. E2 measurement was performed with the reconstituted solution using VARIOSKAN FLASH (Thermo Electron Corporation).

### 2.7 Cumulus expansion assessment

Cumulus expansion was assessed according to a subjective scoring system, cumulus expansion index, on a scale of 0–4.^25^ Zero indicates no expansion, 1 indicates the minimal expansion, 2 indicates the outer cumulus cell layers expansion, 3 indicates all cumulus cell layers expansion except the corona radiata, and 4 indicates all cumulus cell layers expansion including the corona radiata. The referred value was obtained by calculating the average value of cumulus expansion index score in each group. The assessment was blinded to remove bias in four replicate experiments (C‐C: *n* = 59, H‐C: *n* = 56, A‐C: *n* = 50, H‐H: *n* = 44, H‐A: *n* = 52).

### 2.8 Assessment of nuclear maturation

Oocytes were separated from cultured COCs, and the nuclear status was assessed as described previously.^24^, ^26^ GVBD rate after pre‐IVM was analyzed by the oocytes cultured in pre‐IVM for 12 h (Control: *n* = 50, Holo‐TF: *n* = 32, Apo‐TF: *n* = 49). Degenerate rate, GV rate, GVBD rate, and MII rate after IVM were analyzed by the oocytes cultured in IVM for 48 h (C‐C: *n* = 40, H‐C: *n* = 40, A‐C: *n* = 40, H‐H: *n* = 41, H‐A: *n* = 41). Oocytes were fixed with acetic acid/ethanol (1:3) for 48 h and stained with aceto‐lacmoid before examination under a phase‐contrast microscope (400×; Olympus CH, Olympus) for evaluation of their chromatin configuration.

### 2.9 Pre‐IVM and IVM of porcine COCs

For the pre‐IVM and IVM of COCs, oocytes having an evenly granulated cytoplasm with at least four layers of unexpanded cumulus cells were selected by microscopic examination and washed three times. Twenty COCs were cultured in 100 μL of mNCSU37 medium at 39°C in a humidified incubator (95% air, 5% CO_2_). The mNCSU37 medium was supplemented with 2 mM hypoxanthine, 10% (v/v) FCS (Gibco BRL) and 7 mM taurine (Sigma‐Aldrich) and used as basic medium for pre‐IVM and IVM.

In this study, we detected that TF concentration in follicular fluid collected from large antral follicle was 13.5 mg/mL. In 10% FCS of mNCSU37 medium, 0.5 mg/mL TF was included (data not shown), thus, we added the 13.0 mg/mL of Holo‐ and Apo‐TF to pre‐IVM medium and IVM medium in this study.

**FIGURE 1:**
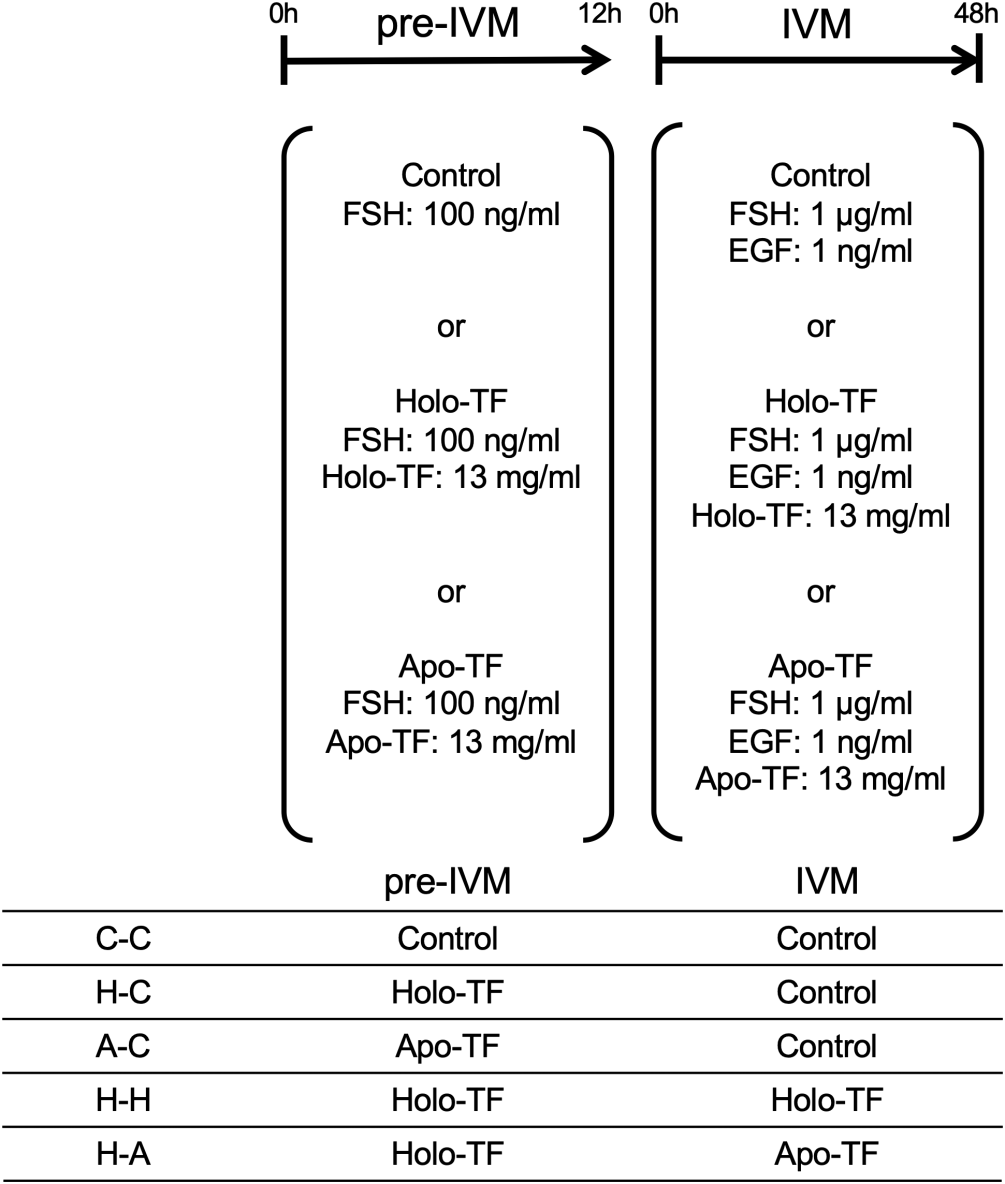
Scheme of pre‐IVM and IVM COCs were cultured in pre‐IVM medium without or with TF for 12 h. Precultured COCs were then cultured in IVM medium without or with TF for 48 h. After 48 h of IVM culture, COCs were cocultured with sperm, and fertilized oocytes were cultured for 7 days to produce blastocyst. COCs, cumulus–oocyte complexes; IVM, in vitro maturation; TF, transferrin.

To investigate the effects of Holo‐TF during pre‐IVM on the cumulus cells functions of COCs and meiotic resumption of oocytes, COCs were cultured in Control, Holo‐TF, and Apo‐TF group for 6, 10, or 12 h. Control group was cultured in maturation medium supplemented with low dose (100 ng/mL) of highly purified FSH. Holo‐TF or Apo‐TF group were cultured in pre‐IVM medium supplemented with low dose (100 ng/mL) of highly purified FSH and either 13 mg/mL Holo‐TF or 13 mg/mL Apo‐TF (Figure 1). After pre‐IVM, culture medium was used for E2 concentration (12 h). Precultured COCs were used for total RNA isolation (6 h), detection of PCNA‐positive cells (10 h), morphology analysis (12 h), assessment of meiotic resumption of oocytes (12 h), and subsequent IVM.

To examine the effect of Holo‐TF during IVM on the cumulus cells functions or oocyte maturation, COCs were cultured in Control, Holo‐TF, or Apo‐TF group for 12 h in advance (pre‐IVM). After pre‐IVM, COCs were cultured in Control, Holo‐TF, and Apo‐TF group for 3 h or 48 h (IVM). Control group of IVM medium was supplemented with high dose (1 μg/mL) of highly purified FSH and 1 ng/mL EGF. Holo‐TF or Apo‐TF group were cultured in IVM medium supplemented with high dose (1 μg/mL) of highly purified FSH and 1 ng/mL EGF either 13 mg/mL Holo‐TF or 13 mg/mL Apo‐TF (Figure 1). Pre‐IVM + IVM culture performed five groups by the combination of pre‐IVM and IVM culture group (Control–Control: C‐C, Holo‐TF‐Control: H‐C, Apo‐TF‐Control: A‐C, Holo‐TF‐Holo‐TF: H‐H, and Holo‐TF‐Apo‐TF: H‐A). After pre‐IVM + IVM, COCs were used for the expression of COC expansion‐related genes (3 h), the assessment of COC expansion (48 h), the measurement of degenerate rate (48 h), GV rate (48 h), GVBD rate (48 h), and MII rate (48 h) and subsequent in vitro fertilization (IVF).

### 2.10 In vitro fertilization (IVF)

In vitro fertilization was performed according to our previous report.^5^ Briefly, cumulus cell‐free oocytes were washed three times with fertilization medium, that is, modified Tris‐buffered medium (mTBM) supplemented with 0.1% (w/v) BSA (fraction V, A 7888: Sigma Chemical Co) and 1 mM caffeine (Sigma Chemical Co). After washing, 20 oocytes were placed in 50 μL of the fertilization medium that had been covered with mineral oil in a 35 × 10‐mm^2^ polystyrene culture dish (Falcon). The dishes were kept in an incubator for about 30 min until spermatozoa were added for fertilization. Fresh spermatozoa from a Duroc boar were purchased from Swinegenetics, washed by centrifugation at 700 *g* for 5 min in washing medium, that is, mTBM supplemented with 0.1% (w/v) BSA. The sperm pellet was resuspended and precultured for 90 min in preculture medium, that is, mTBM supplemented with 10% (v/v) fetal calf serum and 1 mM caffeine. The concentration of spermatozoa during precultivation was 2 × 10^8^ cell/mL. The precultured spermatozoa were diluted to 2 × 10^6^ cells/mL in the fertilization medium, and 50 μL of this sperm suspension was added to 50 μL of the fertilization medium that contained oocytes (final concentration of sperm, 10^6^ cells/mL). Oocytes were cocultured with spermatozoa at 39°C in an atmosphere of 5% CO_2_ in air for 6 h. The mTBM used for IVF was essentially the same as that used by Abeydeera and Day.^27^ Because the pH of basic mTBM just after preparation is about 9.8–10, the final IVF medium was kept in the incubator (an atmosphere of 5% CO_2_ in air at 39°C) for 18–24 h to stabilize the pH to 7.2–7.3 before use (C‐C: *n* = 60, H‐C: *n* = 58).

### 2.11 In vitro culture (IVC)

After sperm–oocyte coincubation, putative zygotes were cultured according to our previous report.^5^ After the gametes were coincubated for 6 h, the putative zygotes were washed three times and transferred into IVC medium. The day of insemination was defined as Day 0. The basic IVC medium was mNCSU37 medium containing 0.4% BSA (fraction V, A 8022: Sigma Chemical Co). Embryos were cultured in IVC medium supplemented with 0.17 mM sodium pyruvate (Sigma Chemical Co) and 2.73 mM sodium lactate from Day 0 to Day 2, then in IVC medium with 5.55 mM D‐glucose, as previously described,^5^ from Day 2 to Day 6. At 12 and 144 h after IVF, the proportions of pronuclear formation and blastocyst formation, respectively, were evaluated. The cleaved embryos were examined by a phase‐contrast microscope at Day 2 in culture. At Day 7, embryos were fixed and placed on slides with drops of mounting media with DAPI (C‐C: *n* = 60, H‐C: *n* = 58).

### 2.12 Statistical analyses

All data were obtained from three or four replications for comparison, and are presented as the mean ± SEM. The degenerate rate, GV rate, GVBD rate, and MII rate were calculated as the number of oocytes showing degenerate, GV, GVBD, and MII divided by the total oocytes number. The blastocyst rate was calculated by dividing the number of embryos that reached the blastocyst stage by the number of oocytes extracted first polar body. All statistical analyses were preformed using Statview software (Avacus Concepts). All percentage data were subjected to arcsine transformation before analysis. All the datasets (mRNA expression, TF concentration, iron concentration, E2 concentration, rate of PCNA‐positive cumulus cells, cumulus expansion index, MII rate, blastocyst rate) were carried out by Student's t‐test, one‐way ANOVA followed by Fisher's least significant difference. *p* < 0.05 was considered statistically significant.

## 3 RESULTS

### 3.1 Tf mRNA expression in cumulus cells, TF amount in follicular fluid, and TFR1 localization in granulosa cells and cumulus cells collected from small, medium, and large antral follicles

**FIGURE 2:**
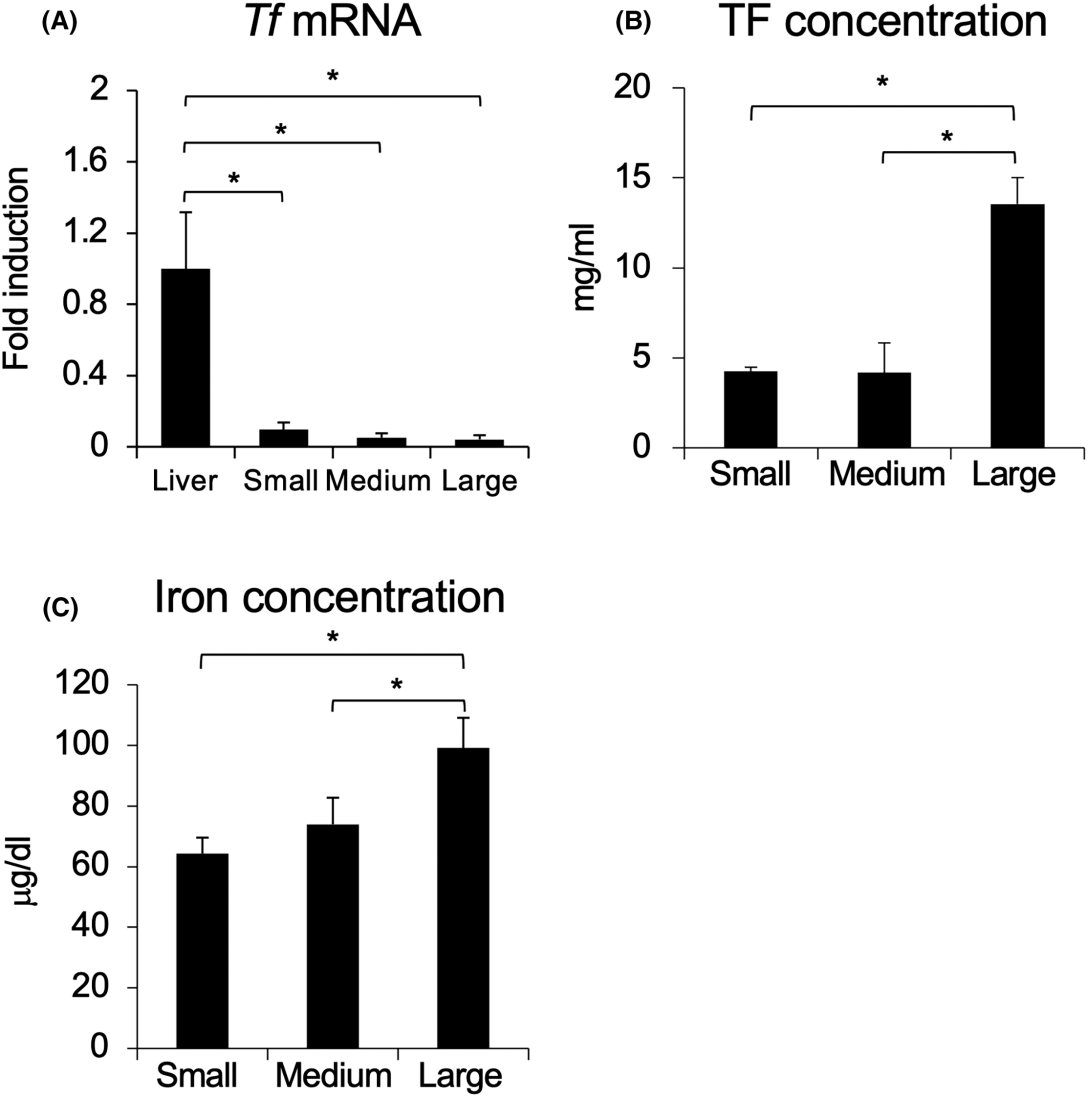
*Tf* mRNA expression in COCs and TF and iron concentration in follicular fluid collected from small, medium, and large antral follicles. COCs and follicular fluid were collected from small (1–3 mm), medium (4–8 mm), and large (8 mm<) antral follicles. *Tf* mRNA expression in COCs were analyzed by real‐time RT‐PCR. TF and iron concentration in follicular fluid were analyzed by ELISA and iron assay, respectively. (A) *Tf* mRNA expression in COCs collected from small, medium, and large antral follicles and in liver. (B) TF concentration in follicular fluid collected from small, medium, and large antral follicles. (C) Iron concentration in follicular fluid collected from small, medium, and large antral follicles. Values are mean ± SEM of three independent experiments (*n* = 3). * Significant differences were observed (*p* < 0.05). COCs, cumulus–oocyte complexes; TF, transferrin.

**FIGURE 3:**
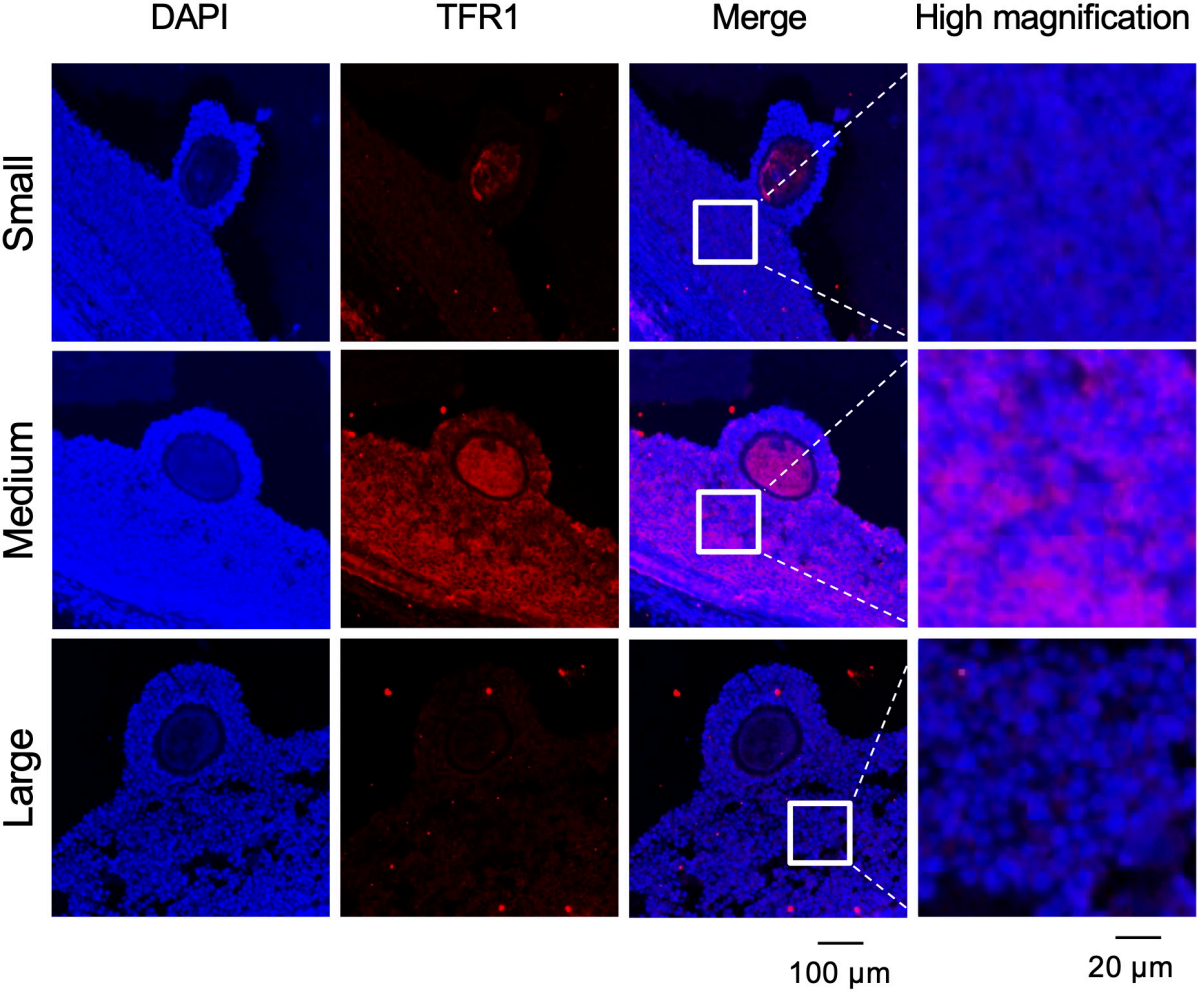
TFR1 localization in granulosa and cumulus cells collected from small, medium, and large antral follicles. Follicles were collected from small (1–3 mm), medium (4–8 mm), and large (8 mm<) antral follicles. TFR1 localization in follicle was visualized by immunofluorescence staining using TFR1 antibody (red color). Nuclear staining was visualized with DAPI (blue color). Scale bar are 100 and 20 μm. TFR1, TF receptor.

Figure 2 shows the kinetic change in *Tf* mRNA expression in cumulus cells collected from small (1–3 mm), medium (4–7 mm), and large antral follicles (bigger than 8 mm). As a positive control, the expression of *Tf* mRNA in the liver was compared with that in cumulus cells. *Tf* mRNA expression levels in cumulus cells of COCs collected from small, medium, and large antral follicles were significantly lower than that in liver (Figure 2A). On the other hand, although the TF amount in follicular fluid collected from small and medium antral follicles were low levels (small: 4.26 ± 0.22 mg/mL, medium: 4.17 ± 1.65 mg/mL), the amount in large antral follicles was significantly larger than that in small and medium antral follicle (large: 13.55 ± 1.46 mg/mL; Figure 2B). Likewise, iron concentration was low in follicular fluid collected from small and medium follicles but were much higher in large follicle (small: 64.31 ± 5.26, medium: 73.97 ± 8.77 μg/dL, and large: 99.15 ± 9.96 μg/dL; Figure 2C). TFR1 protein localization in small and large antral follicles was low level, whereas the red signal showing TFR1 protein localization in medium follicles was higher, and the signals were in both granulosa cells and cumulus cells (Figure 3).

### 3.2 Effect of pre‐IVM with Holo‐TF on meiotic resumption of oocytes, expression on follicular development makers in cumulus cells and E2 concentration in cultured medium after pre‐IVM

**FIGURE 4:**
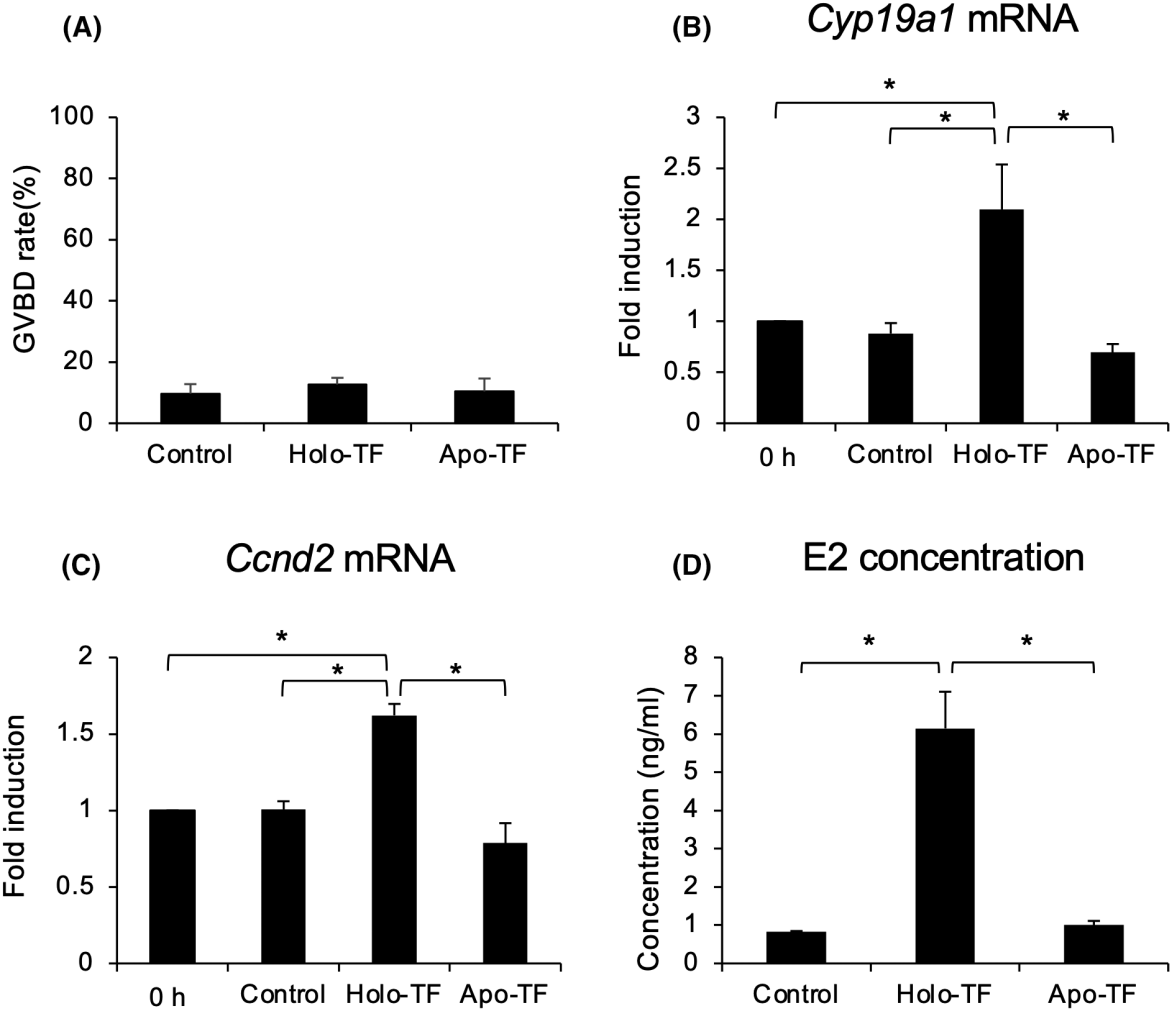
Effects of pre‐IVM with Holo‐TF on GVBD rate, the expression of *Cyp19a1* and *Ccnd2* in cumulus cells, and E2 concentration in cultured medium. COCs collected from medium antral follicles (4–7 mm) were cultured in pre‐IVM medium without or with Holo‐TF or Apo‐TF for 6 h (*Cyp19a1* and *Ccnd2*) or 12 h (GVBD rate and E2). After cultivation, GVBD rate of precultured oocytes (A) was assessed, and expression of *Cyp19a1* (B) and *Ccnd2* (C) mRNA in precultured cumulus cells were analyzed by real‐time RT‐PCR. E2 concentration (D) in preculture medium was analyzed by ELISA. 0 h; *Cyp19a1* or *Ccnd2* mRNA expression in COCs just after collected from medium antral follicles. Control; COCs were cultured without TF. Holo‐TF; COCs were cultured with Holo‐TF. Apo‐TF; COCs were cultured with Apo‐TF. Values are mean ± SEM of three independent experiments (*n* = 3). * Significant differences were observed (*p* < 0.05). COCs, cumulus–oocyte complexes; IVM, in vitro maturation; TF, transferrin.

During the follicular development, meiotic resumption of oocytes was suppressed by hypoxanthine, a physiological inhibitor of oocyte meiotic resumption in pigs.^28^ Our previous study showed that the addition of 2 mM hypoxanthine significantly suppressed spontaneous oocyte meiotic maturation in pigs^29^; therefore, in this study, COCs were cultured in pre‐IVM medium supplemented with 2 mM hypoxanthine for 12 h, and the effect of pre‐IVM on meiotic resumption of oocytes was investigated. The GVBD rate of oocyte cultured in control was almost 10% and the value was similarly low even when Holo‐TF or Apo‐TF was added to control medium (Figure 4A). These low GVBD rates in control, Holo‐TF, and Apo‐TF groups were similar values to previous reports.^28^, ^29^, ^30^

To mimic the follicular development environment, COCs were cultured in pre‐IVM medium without (Control) or with Holo‐TF for 6 or 12 h. As a negative control, COCs were cultured in pre‐IVM medium with Apo‐TF for 6 or 12 h. After pre‐IVM, the expression level of follicular development marker (*Cyp19a1* and *Ccnd2*) mRNA in cumulus cells (6 h) and the E2 concentration in cultured medium (12 h) were measured. When COCs were cultured in pre‐IVM medium for 6 h without TF (Control), the expression levels of *Cyp19a1* and *Ccnd2* mRNA were low level and these expression levels were comparable to preculture levels (0 h), whereas the expression levels in cumulus cells of COCs cultured for 6 h in pre‐IVM medium with Holo‐TF were significantly higher than those of 0 h and the Control. Cultivation with Apo‐TF significantly reduced these high *Cyp19a1*, and *Ccnd2* mRNA expression levels obtained by cultivation with Holo‐TF (Figure 4B,C). The E2 concentration in follicular fluid of the Holo‐TF treatment group was higher than that in Control (Holo‐TF: 6.15 ± 0.96 ng/mL, Control: 0.83 ± 0.01 ng/mL); however, Apo‐TF cultivation significantly reduced the E2 concentration in the Holo‐TF group (Figure 4D).

### 3.3 Effect of pre‐IVM with Holo‐TF on proliferation of cumulus cells of COCs

**FIGURE 5:**
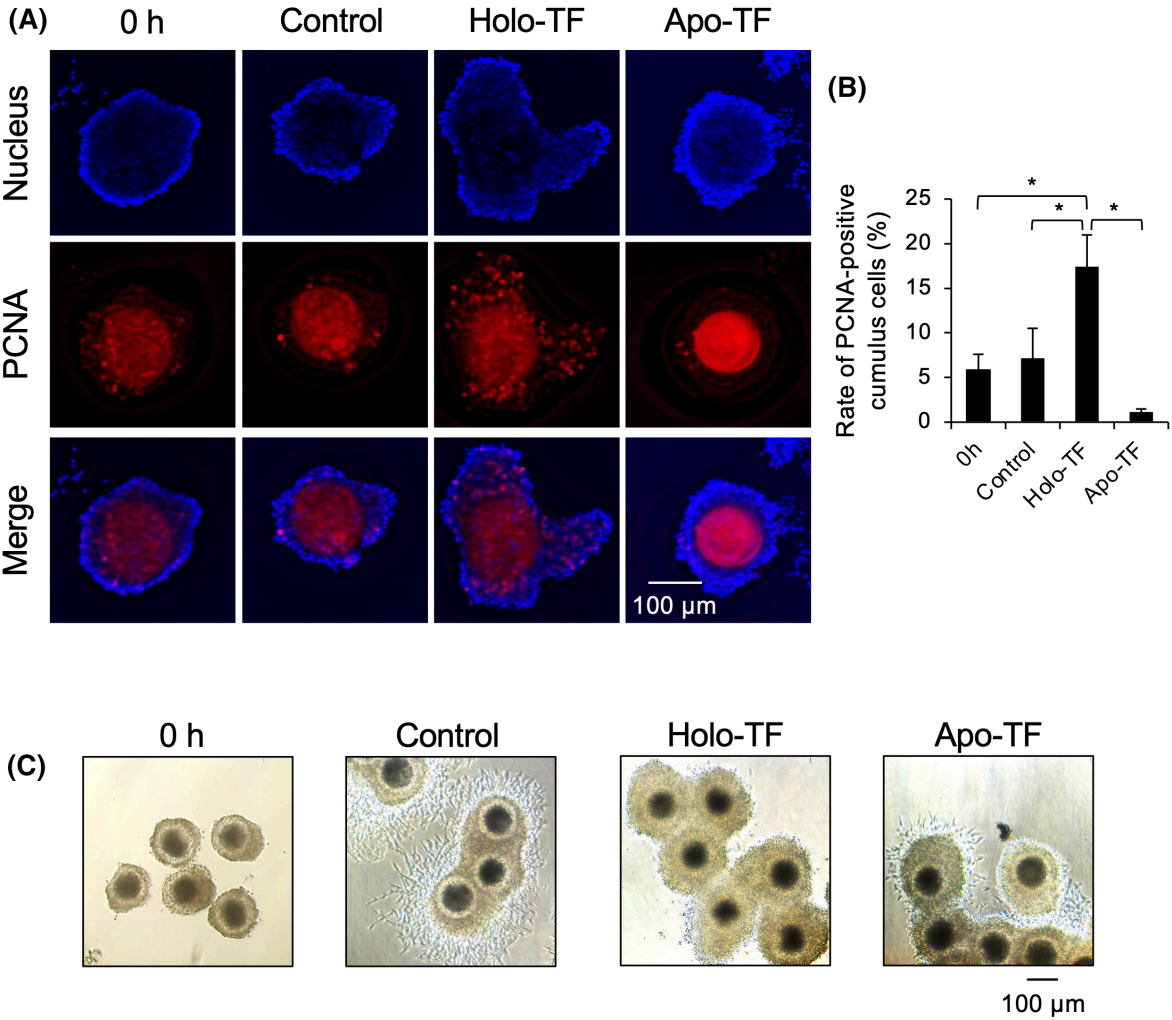
Effect of pre‐IVM with Holo‐TF on COCs. COCs collected from medium antral follicles (4–7 mm) were cultured in pre‐IVM medium without or with Holo‐TF or Apo‐TF for 10 h or 12 h. After 10 h, PCNA‐positive cumulus cells were visualized by immunofluorescence staining using PCNA antibody (red color). Nuclear staining was visualized with DAPI (blue color). After 12 h, morphology of COCs was observed by phase contrast microscopy. (A) Effect of pre‐IVM with Holo‐TF on proliferation of cumulus cells. Red signals showed the localization of PCNA‐positive cumulus cells. Scale bar is 100 μm. (B) Effect of pre‐IVM with Holo‐TF on the rate of PCNA‐positive cumulus cells of COCs. (C) Effect of pre‐IVM with Holo‐TF on the morphology of COCs. Scale bar is 100 μm. 0 h; COCs just after collected from medium antral follicles. Control; COCs were cultured without TF. Holo‐TF; COCs were cultured with Holo‐TF. Apo‐TF; COCs were cultured with Apo‐TF. Values are mean ± SEM of three independent experiments (*n* = 3). * Significant differences were observed (p<0.05). COCs, cumulus–oocyte complexes; IVM, in vitro maturation; TF, transferrin.

In this experiment, we examined the effect of pre‐IVM with Holo‐TF on the proliferation of cumulus cells of COCs. After pre‐IVM, PCNA‐positive cumulus cells were detected. A lot of fluorescence intensity indicating PCNA‐positive cumulus cells of COCs cultured without Holo‐TF for 10 h were low and comparable to those of cumulus cell of COCs just after collection from medium follicles (0 h); however, the low level was significantly increased by cultivation with Holo‐TF in pre‐IVM medium. Cultivation of COCs in Apo‐TF‐contained medium decreased the number of PCNA‐positive cumulus cells compared with that of COCs in Holo‐TF‐contained medium (Figure 5A,B).

Before cultivation in pre‐IVM medium, the cumulus cells of COCs collected form medium antral follicle were intact, whereas, after cultivation without TF, the outer layer of the cumulus cells of COCs was dropped onto the culture plate, and the morphology was changed to that of flattened cells. The addition of Holo‐TF to pre‐IVM medium maintained intact cumulus cells and the layer of the cumulus cells was increased, whereas the outer layer of the cumulus cells was changed to that of flattened cells when COCs were cultured with Apo‐TF (Figure 5C).

### 3.4 Effect of IVM with Holo‐TF on COC expansion, oocyte maturation, and oocyte developmental competence of COCs

**FIGURE 6:**
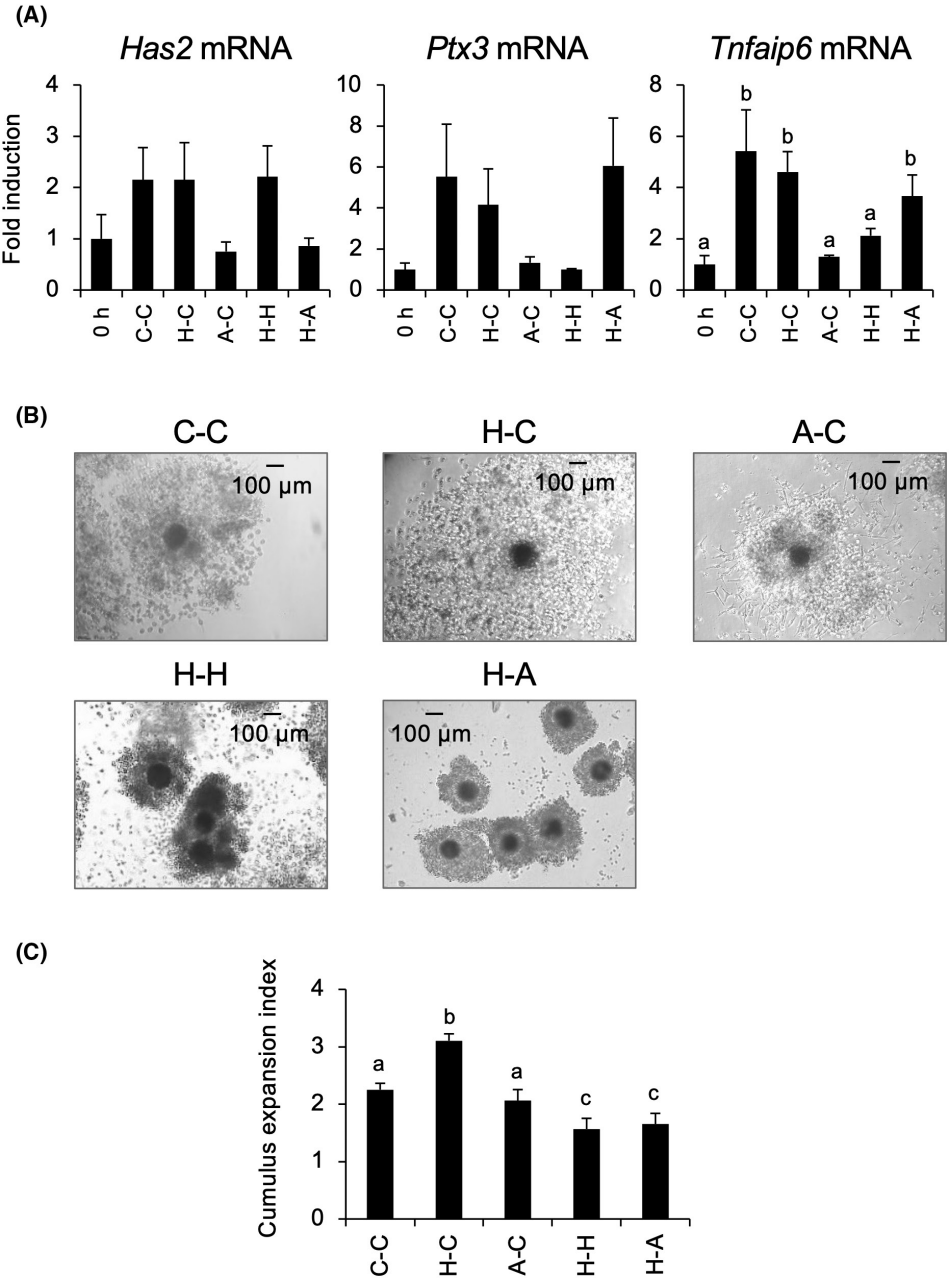
Effects of IVM with Holo‐TF on the expression of COC expansion‐related genes and Cumulus expansion index. COCs collected from medium antral follicles (4–7 mm) were cultured in pre‐IVM medium without or with Holo‐TF or Apo‐TF for 12 h and then were cultured in IVM medium without or with Holo‐TF or Apo‐TF for 3 h (A) and 48 h (B,C). (A) After cultivation, *Has2* (left), *Ptx3* (middle), or *Tnfaip6* (right) mRNA expression in cultured cumulus cells were analyzed by real‐time RT‐PCR. (B,C) Morphology of COCs was observed by inverted microscopy (B) and cumulus expansion index was analyzed (C). 0 h; *Has2*, *Ptx3* or *Tnfaip6* mRNA expression in COCs just after collected from medium antral follicles. C‐C; COCs were cultured in pre‐IVM medium without TF, and then cultured in IVM medium without TF. H‐C; COCs were cultured in pre‐IVM medium with Holo‐TF, and then cultured in IVM medium without TF. A‐C: COCs were cultured in pre‐IVM medium with Apo‐TF, and then cultured in IVM medium without TF. H‐H; COCs were cultured in pre‐IVM medium with Holo‐TF, and then cultured in IVM medium with Holo‐TF H‐A; COCs were cultured in pre‐IVM medium with Holo‐TF, and then cultured in IVM medium with Apo‐TF. Scale bar is 100 μm. Values are mean ± SEM of three independent experiments (*n* = 3). a‐c, Significant differences were observed among each culture group (*p* < 0.05). COCs, cumulus–oocyte complexes; IVM, in vitro maturation; TF, transferrin.

To examine whether Holo‐TF is necessary for COC expansion, oocyte maturation, and the developmental competence of oocytes of COCs during IVM, COCs were cultured in pre‐IVM medium without or with Holo‐TF or Apo‐TF for 12 h, after which the cultured COCs were cultured in IVM medium without or with Holo‐TF or Apo‐TF for 3 h. Expression levels of *Has2* and *Ptx3* mRNA did not differ significantly among the treatment groups, whereas *Tnfaip6* mRNA expression was significantly increased by cultivation in C‐C, H‐C, and H‐A treatments compared with that in before culture (0 h; Figure 6A).

In the C‐C group, the outer layer of the cumulus cells of COCs was expanded, however, the inside of the cumulus cells was not fully expanded. In the H‐C group, both the outer and inner layers of the cumulus cells were fully expanded; however, the A‐C treatment induced the expansion of only the outer layer of the cumulus cells. In the H‐H and H‐A groups, cumulus expansion was also not fully induced (Figure 6B). Cumulus expansion index in the H‐C groups were significantly larger than those in the C‐C group (C‐C: 2.3 ± 0.1 vs. H‐C: 3.1 ± 0.1); however, cultivation with A‐C, H‐H, or H‐A treatment resulted in significantly smaller diameters than those in the H‐C group (Figure 6C).

**FIGURE 7:**
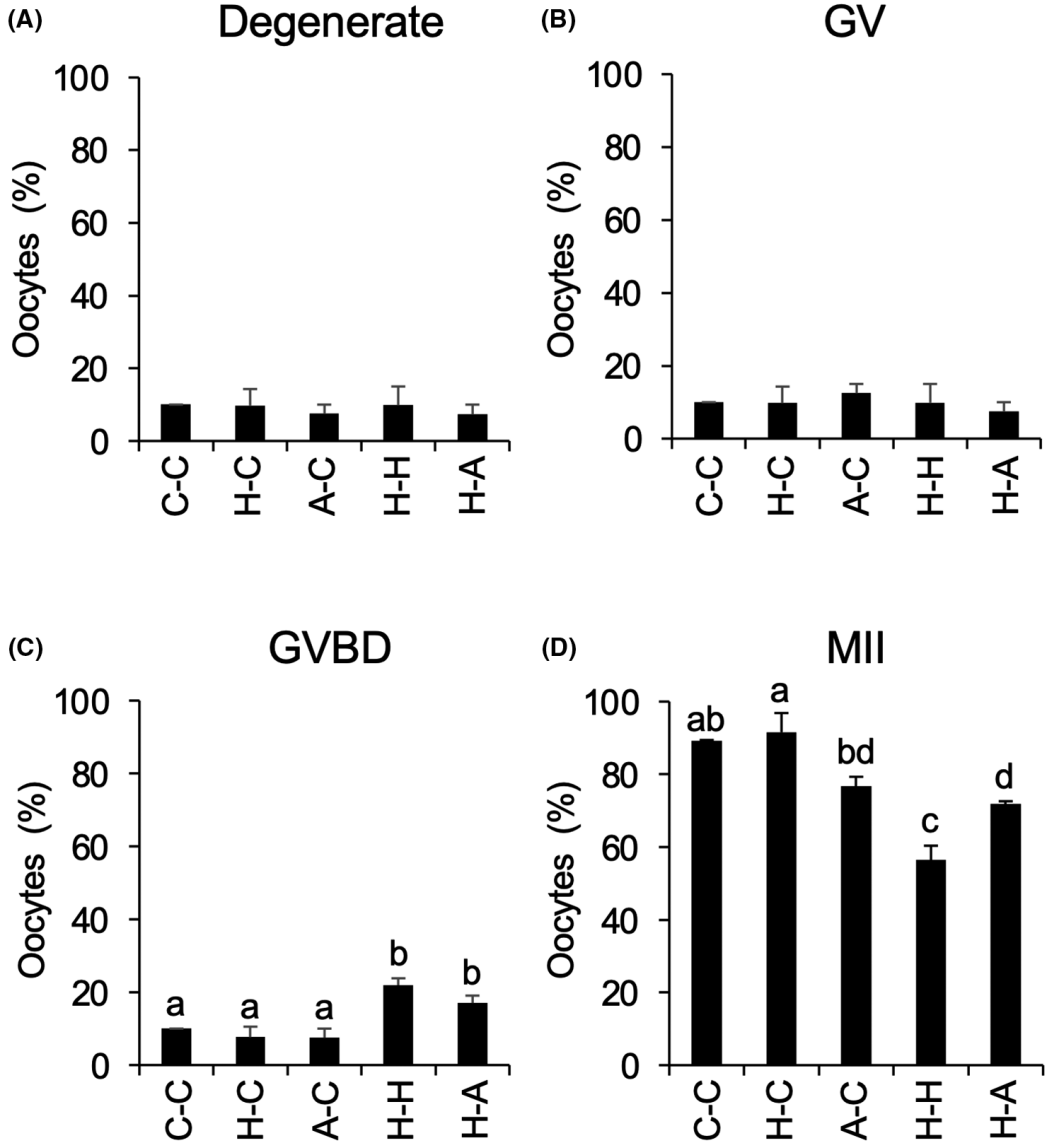
Effects of IVM with Holo‐TF on the oocyte maturation. COCs collected from medium antral follicles (4–7 mm) were cultured in pre‐IVM medium without or with Holo‐TF or Apo‐TF for 12 h and then were cultured in IVM medium without or with Holo‐TF or Apo‐TF for 48 h. Nuclear status of oocytes was evaluated by chromatin configurations ((A) Degenerate rate; (B) GV rate; (C) GVBD rate and (D) MII rate). 0 h; Has2, Ptx3, or Tnfaip6 mRNA expression in COCs just after collected from medium antral follicles. C‐C; COCs were cultured in pre‐IVM medium without TF, and then cultured in IVM medium without TF. H‐C; COCs were cultured in pre‐IVM medium with Holo‐TF, and then cultured in IVM medium without TF. A‐C: COCs were cultured in pre‐IVM medium with Apo‐TF, and then cultured in IVM medium without TF. H‐H; COCs were cultured in pre‐IVM medium with Holo‐TF, and then cultured in IVM medium with Holo‐TF. H‐A; COCs were cultured in pre‐IVM medium with Holo‐TF, and then cultured in IVM medium with Apo‐TF. Values are mean ± SEM of three independent experiments (*n* = 3). a‐c, Significant differences were observed among each culture group (*p* < 0.05). COCs, cumulus–oocyte complexes; IVM, in vitro maturation; TF, transferrin.

After pre‐IVM, COCs were cultured in IVM medium at Control, Holo‐TF, and Apo‐TF group and the degenerate rate, GV rate, GVBD rate, and MII rate was measured. The degenerate rate, GV rate were low in all groups, whereas GVBD rate in H‐H group and H‐A group were significantly high compared with that in C‐C, H‐C, and A‐C groups (Figure 7A–C). In the C‐C, H‐C, A‐C, and H‐A groups, the MII rate were relatively high, almost 80%; however, the rate in the H‐H group was significantly lower by comparison (Figure 7D).

**FIGURE 8:**
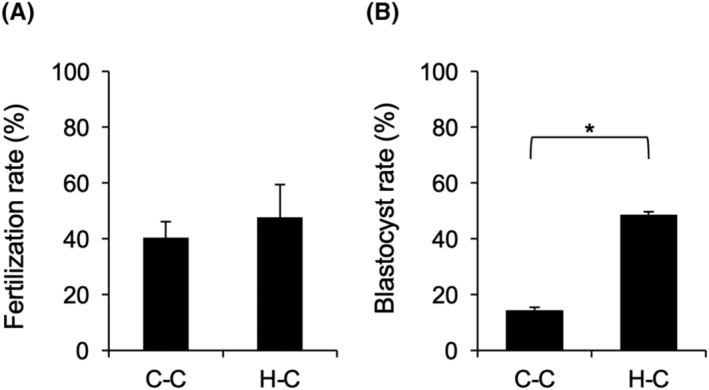
Effect of pre‐IVM with Holo‐TF on fertilization rate (A) and blastocyst rate (B). COCs collected from medium antral follicles (4–7 mm) were cultured in pre‐IVM medium without or with Holo‐TF for 12 h, and then were cultured in IVM medium without Holo‐TF and Apo‐TF for 48 h. After cultivation, sperm and oocytes were coincubated in IVF medium, and then cultured for 7 days. C‐C; COCs were cultured in pre‐IVM medium without TF, and then cultured in IVM medium without TF. H‐C; COCs were cultured in pre‐IVM medium with Holo‐TF, and then cultured in IVM medium without TF Values are mean ± SEM of three independent experiments (*n* = 3). * Significant differences were observed (*p* < 0.05). COCs, cumulus–oocyte complexes; IVM, in vitro maturation; TF, transferrin.

After insemination, the fertilization rate in the C‐C and H‐C group was almost 40%, and the blastocyst rate in the C‐C group was low, but H‐C treatment significantly and dramatically increased the rate (C‐C; 14.4 ± 3.2% vs. H‐C; 48.7 ± 3.9%; Figure 8A,B).

## 4 DISCUSSION

Oocyte maturation occurs during two different phases: the follicular developmental phase and the ovulatory phase. In the former, COCs proliferate and accumulate the energy and nutrition needed for embryogenesis.^31^, ^32^ In the later, cumulus expansion and meiotic maturation of oocytes occur.^33^ It is well recognized that matured oocytes formed through these two phases have high developmental competence.^4^, ^34^, ^35^ COCs cultured by the IVM are usually recovered from developing follicles (4–7 mm diameter), however, COCs are conventionally cultured in the high dose of FSH condition where the ovulation phase during IVM is mimicked.^36^, ^37^, ^38^, ^39^ Thus, it is reasonable to adapt a novel IVM method that is consistent with the environment of both the follicular development phase and the ovulatory phase.

Although there are few reports about TF expression in the ovary, the localization of TF has been observed in granulosa cells in antral follicles during follicular development.^22^ In this study, we also showed the expression of *Tf* mRNA in developing antral follicles, though levels extremely low compared to those in liver. On the other hand, we showed for the first time that TF concentration in large antral follicles was 13.5 mg/mL, more than threefold the approximately 4 mg/mL found in both serum and liver.^40^ Since we confirmed that *Tf* mRNA, TF protein expression, and iron content in liver were significantly upregulated after eCG injection in mice (data not shown), we inferred that TF‐Fe^3+^ was supplied by the liver via blood vessels to induce follicular development. In hepatocytes, TRF1 is known to be recycled when it binds to TF,^41^ whereas in granulosa and cumulus cells of large antral follicle, its expression is low, suggesting that the TFR1 recycling is suppressed in granulosa cells and cumulus cells of large antral follicle. The mechanism by which TFR1 recycling does not occur in large antral follicle is unknown. If downregulation of TFR1 can induce during IVM, it may be possible to increase the developmental competence of oocytes by IVM.

Numerous studies showed that FCS supplementation to conventional IVM medium enhanced oocyte maturation during conventional IVM in mice,^42^ rabbits,^43^ bovine,^44^ and pigs.^45^ A lot of previous reports have been conducted on appropriate FCS concentrations to induce sufficient maturation of oocytes in bovine^44^, ^46^ and pigs.^47^, ^48^ As a result, it is now common to supplement maturation medium with FCS at a concentration of 10%. In maturation medium that concentration, we confirmed that almost 400 μg/mL of TF was contained (data not shown). In this study, we used a quite high concentration of Holo‐TF (13.5 mg/mL) as pre‐IVM medium based on the TF concentration of follicular fluid collected from large antral follicles. As a result, the combined cultivation with Holo‐TF in pre‐IVM medium and without Holo‐TF in IVM medium significantly upregulated the proliferative activity and E2 production ability of cumulus cells during pre‐IVM, and increased COC expansion, which resulted in the increment of the blastocyst rate after subsequent IVM. Therefore, these results indicated that culturing at a high TF concentration during pre‐IVM is quite important for improving the developmental competence of oocytes in pigs.

In this study, since 13.5 mg/mL of TF was contained in large antral follicle, we added this TF concentration to the pre‐IVM and IVM medium in which COCs were cultured and examined the effect on COC and oocyte function. In our preliminary experiments, we confirmed that the lower concentration of TF used in this study had no effect on developmental competence of oocytes. Thus, in this experiment, we added physiological concentration of TF (13.5 mg/mL) to pre‐IVM and IVM medium. As the results, we succeeded in improving the blastocyst rate up to 40%. In this experiment, we showed that TF concentration in large antral follicle is threefold high compared with medium antral follicle, whereas Fe concentration is 20% higher in large antral follicle than medium antral follicle. The results indicated that not all Fe is bound to TF in large antral follicle. Previous report showed that two third of TF is not bound to iron,^49^ therefore, it is unlikely that the amount of Fe supplied from TF during oocyte maturation is lower than the concentration of Fe supplied from TF during culture. In this experiment, we suppled 13.5 mg/mL of TF to culture medium during pre‐IVM and/or IVM, however, it is not known whether this TF concentration is optimal for oocyte maturation. In this study, since the information of TF and iron concentration was collected from pig killed in slaughterhouse, hormonal (eCG and hCG) treatment did not conduct. However, our preliminary experiments using mice revealed that the eCG‐primed mice ovaries showed twofold increase compared with non‐eCG‐primed mice ovaries (unpublished data). Moreover, we also showed that the TF in the liver and iron concentration in the blood was increased in response to eCG injection to mice (unpublished data). Collecting the above, it is the possibility that much higher concentration of TF contain in the preovulatory follicle.

To supply TF‐Fe^3+^ during pre‐IVM, culturing with Holo‐TF to pre‐IVM medium, but not in IVM medium (H‐C) was significantly improved COC expansion and developmental competence of oocytes compared to those in the C‐C and A‐C groups. Surprisingly, the continuing addition of Holo‐TF during both pre‐IVM and IVM cultures caused declines in both the COC expansion and fertilization rate. Furthermore, we also revealed that the supplementation of Holo‐TF to maturation medium (without FSH) significantly suppressed spontaneous maturation of porcine oocytes (data not shown). These results implied that iron supplied from TF is necessary in the follicular developmental phase only, and not in the ovulatory phase. In other tissues, previous studies showed that a sufficient amount of iron promotes various cell functions^50^, ^51^; however, excess iron amount causes cell dysfunction by generating reactive oxygen species (ROS).^52^, ^53^ In the ovary, other studies showed that ROS represses COC expansion, oocyte maturation, and oocyte developmental competence.^54^, ^55^ Thus, it is possible that excess iron applied from TF may affect oocyte maturation.

In conclusion, we showed for the first time that TF and iron accumulated in porcine follicular fluid even though *Tf* mRNA was not increased and observed TFR1 protein in granulosa cells and cumulus cells. When COCs were precultured with 100 ng/mL of FSH and Holo‐TF, the E2 production and proliferation activity of cumulus cells increased concomitantly with the upregulation of *Cyp19a1* and *Ccnd2*, respectively. Moreover, cultivation with 1 μg/mL FSH and 1 ng/mL EGF without Holo‐TF after pre‐IVF in medium containing 100 ng/mL FSH and Holo‐TF containing medium enhanced cumulus expansion and oocyte meiotic maturation, which resulted in the increment of the developmental competence of oocytes. From the results of this study, we succeeded in establishing of a novel IVM method in which porcine COCs were cultured in pre‐IVM medium with a low concentration of FSH and Holo‐TF for 12 h followed by culturing in medium containing high concentration of FSH and EGF without Holo‐TF for 48 h.

## FUNDING INFORMATION

This work was supported, in part, by KAKENHI No. 22K19254, 19 K06359 (to Y. Y), No. 21J15868 (to S. T), and 19H03108 (to M. S) from the Japan Society for the Promotion of Science (JSPS).

## CONFLICT OF INTEREST STATEMENT

The authors declare that there is no conflict of interest that could be perceived as prejudicing the impartiality of this report.

## ANIMAL STUDIES

The study was conducted in accordance with the Guideline for the Care and Use of Laboratory Animals of Prefectural University of Hiroshima (approval number 18AO‐006).

## Data Availability

The data presented in this study are available on request from the corresponding author.
